# Detecting spiral wave tips using deep learning

**DOI:** 10.1038/s41598-021-99069-3

**Published:** 2021-10-05

**Authors:** Henning Lilienkamp, Thomas Lilienkamp

**Affiliations:** 1 GFZ Helmholtz Centre Potsdam, Seismic Hazard and Risk Dynamics, Potsdam, 14467 Germany; 2 University of Potsdam, Potsdam, 14469 Germany; 3 Max Planck Institute for Dynamics and Self-Organization, Göttingen, 37077 Germany; 4 German Center for Cardiovascular Research (DZHK), Partner Site Göttingen, Göttingen, 37075 Germany

**Keywords:** Nonlinear phenomena, Computational biophysics, Machine learning

## Abstract

The chaotic spatio-temporal electrical activity during life-threatening cardiac arrhythmias like ventricular fibrillation is governed by the dynamics of vortex-like spiral or scroll waves. The organizing centers of these waves are called wave tips (2D) or filaments (3D) and they play a key role in understanding and controlling the complex and chaotic electrical dynamics. Therefore, in many experimental and numerical setups it is required to detect the tips of the observed spiral waves. Most of the currently used methods significantly suffer from the influence of noise and are often adjusted to a specific situation (e.g. a specific numerical cardiac cell model). In this study, we use a specific type of deep neural networks (UNet), for detecting spiral wave tips and show that this approach is robust against the influence of intermediate noise levels. Furthermore, we demonstrate that if the UNet is trained with a pool of numerical cell models, spiral wave tips in unknown cell models can also be detected reliably, suggesting that the UNet can in some sense learn the concept of spiral wave tips in a general way, and thus could also be used in experimental situations in the future (ex-vivo, cell-culture or optogenetic experiments).

## Introduction

Life-threatening cardiac arrhythmias like ventricular fibrillation are the major cause of morbidity and mortality with sudden cardiac death taking several hundreds of thousands of lives per year. Today, the complex and chaotic dynamics of the underlying electrical excitation patterns in the heart muscle is still not fully understood. However, it is known that spiral or scroll waves are the governing objects which drive the spatio-temporal dynamics during arrhythmias^1–3^. For a deeper understanding of the governing mechanisms and processes which are responsible for the onset and perpetuation of arrhythmias, it is essential to characterize the electrical excitation patterns by tracking and analyzing the spatio-temporal dynamics of the spiral wave tips (or filaments in 3D), since they can be understood as the organizing centers of the waves.

Tracking spiral wave tips is relevant, for example, in the context of controlling^4–6^, and investigating the structure and governing processes during spiral wave chaos^7,8^, analyzing the complexity during cardiac arrhtyhmias^9,10^, or studying creation/termination mechanisms which may also lead to self-termination^11–13^. Spiral or scroll waves can also be found and studied in 3D chemical reactions (scroll waves), like the Belousov–Zhabotinsky reaction^14^, in arrays of non-linear Chua’s circuits^15^ or 2D lattices of van der Pol oscillators^16^.

For this purpose of tracking the positions of spiral wave tips over time, several different methods exist which are based on, for example, differentiating between different states of a grid cell^17,18^, finding intersections of contour lines^19^, finding points of zero normal velocity of isopotential lines^20^, investigating the curvature along isopotential lines^21^, or using the pivot method^22^. Another approach is based on a two-step protocol, where initially a phase is calculated for each point of the spatial domain. This can be done, for instance, by using a Hilbert transformation, or calculating a phase via $\mathrm {atan2}$ based on the membrane potential variable $V_{\mathrm{m}}$ and another secondary variable (or a time delay of $V_{\mathrm{m}}$ instead). Subsequently points with an undefined phase (phase singularities) are associated with the tips of spiral waves^3,23^. However, many of those methods work well in specific situations only. For example, some of them require information about secondary variables, which is mostly the case in numerical simulations, only. Also, most of the methods suffer from the influence of noise, which can not be neglected in experiments, such as optical mapping recordings in ex-vivo Langendorff perfusion experiments^4^.

Recently, R. R. Gurevich and R. O. Grigoriev developed an approach, which is also robust against noise^24^. In this study we follow a different approach using deep learning algorithms, which have been used successfully in the context of dynamical systems, e.g. for modeling^25^ or controlling^26^ dynamical systems. Here, we address the following questions:

1. Can deep neural networks be used to detect positions of spiral waves in general?
2. How robust is such an approach under the influence of noise?
3. How “general” can a deep neural network learn the task of identifying the positions of wave tips: can it identify spiral wave tips for data it has not seen (thus has not been trained on) before (e.g. an unknown numerical cell model, or experimental data)?

The study is structured in the following way: In the next section we briefly describe four different cell models which we investigate. Afterwards, we explain how spiral wave tips are detected using a “conventional” method, in order to generate training data. Then, the architecture and the training procedure of the deep neural network is shown. In the results section, we present the studies which we performed in order to address the three research questions posed before. Finally, we discuss these results in the last section.

## Methodology

In this section, we describe the general approach, more technical details are given in the method section. We start by giving an overview over four different cardiac cell models which we investigate in this study. Afterwards, we describe how episodes of chaotic spiral wave chaos are produced (which is the input data for the UNet), and how phase singularities (tips of the spiral waves) are detected using a conventional method (serving as the ground truth data). We then explain how the UNet is trained and how we evaluate the prediction output of it.

### Overview over the investigated cardiac cell models

The spiral wave dynamics we investigate in this study is described by a system of reaction-diffusion equations Eqs. (1) and (2)

1 $$\begin{aligned} \frac{\partial V_{\mathrm{m}}}{\partial t}= & \, {} D \Delta V_{\mathrm{m}} - \frac{I_{\mathrm{tot}}(V_{\mathrm{m}}, {\mathbf {h}})}{C_{\mathrm{m}}} \, , \end{aligned}$$

2 $$\begin{aligned} \frac{\partial {\mathbf {h}}}{\partial t}= & \, {} {\mathbf {g}}(V_{\mathrm{m}}, {\mathbf {h}}) \, , \end{aligned}$$

where *D* is the diffusion constant, and $C_{\mathrm{m}}$ the membrane capacitance. The exact form of the transmembrane currents $I_{\mathrm{tot}}$ (second term in Eq. (1)) and of ${\mathbf {g}}(V_{\mathrm{m}}, {\mathbf {h}})$ in Eq. (2) is determined by the respective cell model.

**Figure 1:**
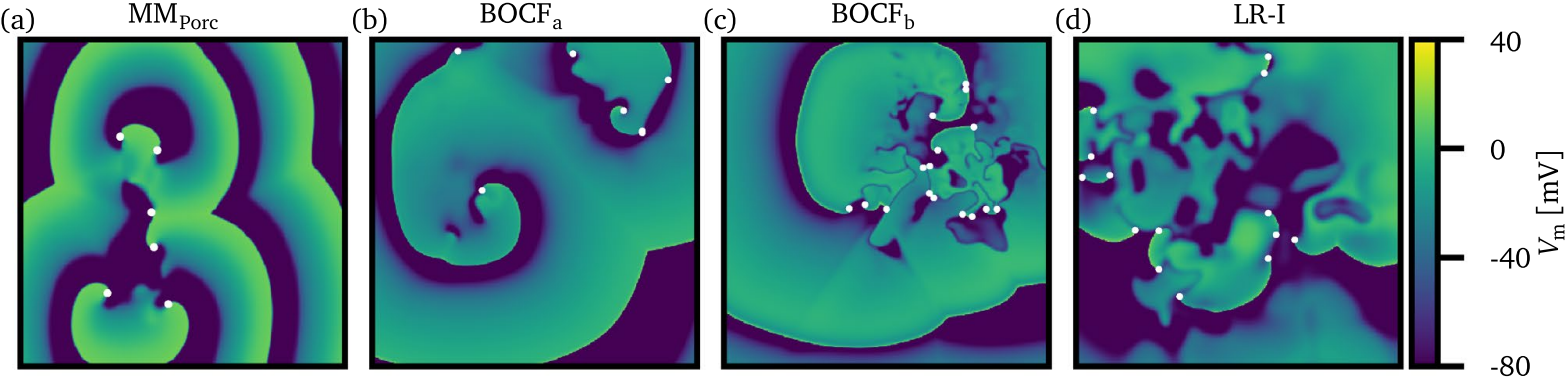
Exemplary snapshots of the membrane potential $\mathrm{V}_{\mathrm{m}}$ are shown for the three-variable minimal model $\mathrm{MM}_{\mathrm{Porc}}$^27^ (**a**), the Bueno-Orovio–Cherry–Fenton model $\mathrm{BOCF}_{\mathrm{a}}$^28^ (**b**) and $\mathrm{BOCF}_{\mathrm{b}}$ (**c**), and the Luo–Rudy-I model LR-I^29^ (**d**), respectively. The organizing centers of spiral waves (phase singularities) are denoted as white dots.

In order to cover a broad range of cardiac cell models, we investigate in this study four different models, ranging from simplified ones to more complex ionic cell models, which describe the electrical action potential dynamics of cardiomyocytes: a three-variable minimal model^27^ describing the porcine electrophysiology (from now on denoted as $\mathrm{MM}_{\mathrm{Porc}}$), the Bueno-Orovio–Cherry–Fenton model^28^ with two different sets of parameters ($\mathrm{BOCF}_{\mathrm{a}}$ and $\mathrm{BOCF}_{\mathrm{b}}$, respectively), and the Luo-Rudy-I model^29^ (LR-I). Details about the cardiac models and parameters are given in the method section. In Fig. 1, exemplary snapshots of the membrane potential $\mathrm{V}_{\mathrm{m}}$ are shown for each model, respectively.

### Preparation of data

For each cell model, we simulated five independent episodes of chaotic spiral wave dynamics with a temporal length of ${1}\,\hbox {s}$ each, and a sampling rate of ${1}\,\hbox {ms}$, respectively. A single spiral wave was initiated and a randomized perturbation of the first variable was added. Afterwards, the initial phase of at least ${2}\,\hbox {s}$ was discarded, in order to ensure that different episodes are independent from each other. This results in total in $5 \times 1000 = 5000$ samples per cell model.

### “Conventional” detection of phase singularities

**Figure 2:**
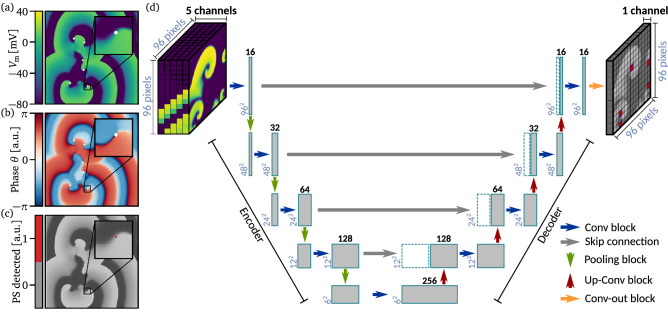
Detection of phase singularities and the architecture of the UNet. An exemplary snapshot ($\mathrm{MM}_{\mathrm{Porc}}$) of the membrane potential $V_{\mathrm{m}}$, and the corresponding phase $\theta$ (as in Eq. (3)) are shown in subplot (**a**), and (**b**), respectively. In subplot (**c**) it is shown how detected phase singularities (spiral wave tips) are marked as red dots of size $1\times 1$ pixel (only the magnified one is visible here). In subplot (**d**), the architecture of the UNet is sketched, where the structure of the denoted Conv blocks, and the Up-Conv blocks is explained in the method section. As an input of the UNet, we use five snapshots of the membrane potential, where the temporal distance between consecutive snapshots is ${5}\,\hbox {ms}$. The output is trained to predict the position of phase singularities (as shown in subplot (**c**)). Note, that for the output, the illustration of the grey spiral waves are not part of the actual data, and are sketched here for the sake of clarity, only.

In order to train the UNet for the purpose of identifying the spatial locations of spiral wave tips, one requires ground truth data such that the UNet can learn where the tips are located. For this task, we use a two step approach: In a first step, the phase at a specific position on the simulation grid and at a given point in time $\theta (t, x, y)$ is computed, based on the (normalized) membrane potential $V_{\mathrm{m}}$ and a second (normalized) dynamical variable *X*, which is chosen for each cell model individually:

3 $$\begin{aligned} \theta (t, x, y) = \mathrm {atan2}(V_{\mathrm{m}}(t, x, y) - V^0_{\mathrm{m}}, \, X(t, x, y) - X^0)\, , \end{aligned}$$

where $V^0_{\mathrm{m}}$ and $X^0$ are reference values whose explicit values are given in the method section. Exemplary snapshots of the membrane potential $V_{\mathrm{m}}$ and the corresponding phase $\theta$ are shown in Fig. 2a,b, respectively.

We associate in the following a spiral wave tip as a phase singularity (PS). Phase singularities are determined by performing path integrals along closed paths $\partial {\mathcal {D}}$ which enclose small domains of $2 \times 2$ pixel of the simulation grid. If the result of such a path integral

4 $$\begin{aligned} \mathrm{PS(t, x, y)} = \left| \frac{1}{2\,\pi }\oint _{\partial {{\mathcal {D}}}}\nabla \theta \mathrm{(t, x, y)}\cdot \mathrm {d}{\mathbf {l}} \right| \end{aligned}$$

is equal to one ($2 \pi$ without the normalization factor), a phase singularity and thus a spiral wave tip has been detected at the specific point in time and space (otherwise the integral is numerically zero). In Fig. 2c, such a phase singularity is shown as a red dot, which can be associated with the spiral wave tip in Fig. 2a (magnified in the black boxes, respectively). In such a way, phase singularities have been detected for each episode of spiral wave dynamics. For the subsequent training procedure of the UNet, the ground truth data consists therefore out of domains containing “zeros” and “ones”, where “ones” indicate the location of a phase singularity.

### Detection of phase singularities via UNet

During the whole study, an input sample consists of five consecutive snapshots ($96 \times 96$ pixel) of the membrane potential $V_{\mathrm{m}}$, which are each separated by 5 ms in time and can thus be considered as a 5-channel image. It is noteworthy that using a temporal sequence of input snapshots here is comparable to the way how the conventional phase can be computed using time delay coordinates. That means, we do not use secondary variables as it was required for the conventional detection of spiral wave tips in the previous section, but we take the past 20 ms of the dynamics into account. The input samples were normalized to values between zero and one to provide optimal conditions for the UNet to learn from the data.

The task at hand is to predict the ground truth position of phase singularities, thus a mask of the spatial domain where a “one” marks the location of a spiral wave tip (as shown in Fig. 2c), from an input sample. The UNet neural network architecture introduced by Ronneberger et al.^30^ is designed to address such a task, i.e. to map multi-channel images of a fixed size to a single-channel image of the same size. The UNet neural network has been developed for the field of biomedical image segmentation where it has outperformed competing state-of-the-art approaches. However, since its development it has also proven useful in various other disciplines such as the geosciences, where it was applied to the problem of pansharpening of satellite images^31^, or electrical engineering, where it was used as a surrogate model for complex physical ray-tracing simulations to model the signal intensity of mobile communication networks^32^. Further applications encompass the detection and removal of artifacts in 2D Sparse Photoacoustic Tomography images^33^, and the segmentation of brain tumor tissue in 3D^34^. The latter study might be of special interest to our field, since it poses a possibility to extend our method from the detection of wave tips (2D) to filaments (3D).

The architecture of the UNet we used throughout this study is depicted in Fig. 2d. More details about the architecture are given in the method section.

For statistically robust statements about the performance of the UNet and to prevent overfitting, we performed a five-fold cross validation for each investigation throughout the whole study: From five episodes of each 1000 samples we created for each cell model, we selected one episode for later validation and performed the training procedure with the remaining 4 episodes (4000 samples), only. This training/validation process was repeated five times, where in each iteration another single episode was left out of the training procedure. The overall performance of the UNet was then computed as a mean over the five obtained validation results.

### Evaluation of the prediction

After the training phase, we use the UNet for predictions of the spatial locations of spiral wave tips. Since the UNet predicts values between 0 and 1, we define a cutoff threshold of $\mathrm{PS}_{\mathrm{cut}}=0.1$ (arbitrarily chosen), that means, if the UNet predicts at a certain location of the grid a value which is larger than $\mathrm{PS}_{\mathrm{cut}}$, we interpret this as a detected phase singularity. In a next step, we compare the predicted locations of spiral wave tips with the ground truth data by using the $F_{\mathrm{score}}$:

5 $$\begin{aligned} F_{\mathrm{score}} = \frac{\text {true positive}}{\text {true positive} + \frac{1}{2}(\text {false positive} + \text {false negative})} \, . \end{aligned}$$

In Eq. (5), if the prediction of the UNet indicates a detected spiral wave tip (thus a value $> \mathrm PS_{\mathrm{cut}}$), we count it as true positive if for the ground truth data we can find a corresponding spiral wave tip in a spatial range of $\pm 2$ pixel, and a temporal range of $\pm ~{5}\,\hbox {ms}$. Likewise, we count a predicted spiral wave tip as false positive, if we cannot find a corresponding one in the ground truth data within the declared temporal and spatial range. Also, if a ground truth spiral wave tip does not have a counterpart in the predicted data, this tip counts as false negative. Thus, if the prediction is accurate, the number of false positives and false negatives is close to zero and therefore the $F_{\mathrm{score}}$ is close to one. With a decreasing prediction quality, the $F_{\mathrm{score}}$ is decreasing from one. The reason we introduce a spatial and temporal uncertainty interval of $\pm 2$ pixel and $\pm ~{5}\,\hbox {ms}$ is that the exact true position of the spiral wave tip cannot be defined uniquely and, for example, in the case of the conventional detection depends on the choice of reference parameters $V_{\mathrm{m}}^0$ and $X^0$ (in Eq. (3)). With this approach, we can distinguish between predicted spiral wave tips which are very close to the true position (which we count as a correct prediction) and predicted tips which are significantly far away from a true spiral wave tip, and are therefore clearly wrong predictions. Still, a spatial uncertainty of $\pm 2$ pixel corresponds in our simulations to approximately 1 mm to 2 mm, which is significantly smaller than typical wave lengths ($\approx {30}$ mm to 100 mm), which is the governing length scale of the system.

## Results

After an exemplary demonstration of how the UNet predicts the location of spiral wave tips in the first subsection, we investigate in this study mainly two aspects: In the following subsection, we show what the influence of noise on the prediction accuracy is, which is measured in terms of the $F_{\mathrm{score}}$. In the subsequent subsection, we investigate whether cross predictions between different cell models can be performed successfully.

### Proof of concept

We trained the UNet for each of the four investigated cell models separately. The $F_{\mathrm{score}}$ was computed for each of the five cases, and average mean values as well as the standard deviation are shown in Table 1.

**Table 1:** Prediction of spiral wave tips. The $F_{\mathrm{score}}$ is shown for the case where the UNet was trained with a single cell model, and the prediction was performed on the same model.

Model	$F_{\mathrm{score}}$
$\mathrm{MM}_{\mathrm{Porc}}$	$0.984 \pm 0.003$
$\mathrm{BOCF}_{\mathrm{a}}$	$0.995 \pm 0.001$
$\mathrm{BOCF}_{\mathrm{b}}$	$0.996 \pm 0.001$
LR-I	0.988 ± 0.007

**Figure 3:**
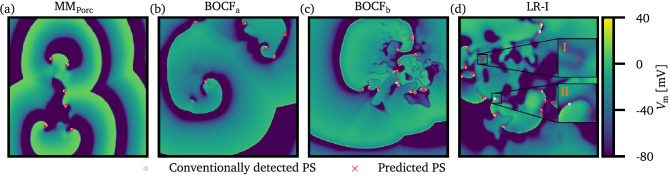
Comparison of spiral wave tips detected by the conventional method (ground truth) and by the UNet. The same exemplary snapshots of the membrane potential $V_{\mathrm{m}}$ as in Fig. 1 are shown here for all four cell models ((**a**–**d**), respectively). The positions of spiral wave tips, which were detected by the conventional method are sketched as light gray dots, whereas the positions predicted by the UNet are shown as red crosses. Note, that the samples shown here did not belong to the training data, which was used during the training phase of the UNet. In general, the prediction of spiral wave tips coincides well with the ground truth data. However, in some cases deviations can be observed: In subplot (**d**), a predicted phase singularity (magnified part I) does not correspond to a ground truth PS. In this case, a phase singularity annihilated shortly ($<{10}\,\hbox {ms}$) before the snapshot was taken. In the second case (magnified part II), a spiral wave front collides with a wave back of a preceding wave, resulting in an abrupt spatial shift of the ground truth phase singularity within a short period of time. In this case, the UNet predicts the position of the PS still at the “former” place. However, within ${10}\,\hbox {ms}$ after the shown snapshot, the ground truth position and the predicted position of the PS coincide again.

Also, for exemplary snapshots of the dynamics, the ground truth position of PS and the predicted positions are shown in Fig. 3. In general, predicted positions of PS coincide well with the ground truth data.

We identified, however, two sources of errors: when PS are created/annihilated the prediction of the present PS can differ from the ground truth data for a short period of time. Also, in the case that a spiral wave front collides with a wave back of another wave, resulting in a sudden shift of the position of the spiral wave tip, can cause a disagreement between ground truth and predicted positions of PS in some cases. Both of these cases are discussed also in Fig. 3d. However, in both cases the deviation between ground truth data and prediction lasts for a short amount of time, only.

### The influence of Gaussian white noise in the prediction

In experimental measurements of spiral wave dynamics (e.g. in the context of ex-vivo Langendorff experiments with optical mapping recordings) the influence of noise is in many cases considerable. That is, why in this section we investigate how robust spiral wave tips can be detected with the UNet, if the input data is noisy.

**Figure 4:**
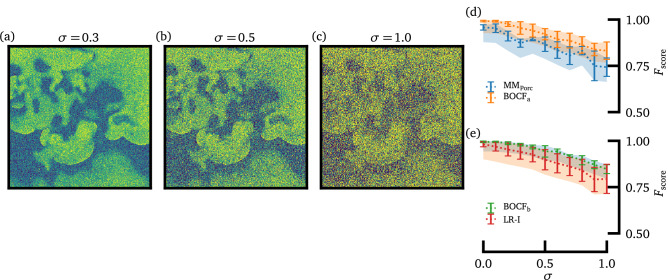
The influence of additive Gaussian white noise on the prediction. In subplots(**a**–**c**), an exemplary snapshot of the membrane potential (LR-I model, same snapshot as in Fig. 1d) is shown for three different values of the standard deviation of the Gaussian distribution $\sigma =0.3, 0.5,$ 1.0, respectively. In subplots (**d**), and (**e**) the $F_{\mathrm{score}}$ is depicted for all four investigated models, depending on $\sigma$.

For this purpose, after normalizing the input data we add white Gaussian noise with the standard deviation $\sigma$ and train the UNet with the noisy data. In Fig. 4a–c exemplary snapshots of noisy input data (LR-I model) are shown for standard deviations of $\sigma =0.3$, $\sigma =0.5$, and $\sigma =1.0$, respectively.

The resulting $F_{\mathrm{score}}$ for a given standard deviation $\sigma$ is shown in Fig. 4d for the $\mathrm{MM}_{\mathrm{Porc}}$ model and the $\mathrm{BOCF}_{\mathrm{a}}$ model, and in (e) for the $\mathrm{BOCF}_{\mathrm{b}}$ model and the LR-I model, respectively. As expected, the prediction performance decreases for all models with increasing $\sigma$, although qualitative differences can be observed between models. For example, it seems that the prediction of spiral wave tips is more robust against the influence of noise in the case of the $\mathrm{BOCF}_{\mathrm{a}}$ and $\mathrm{BOCF}_{\mathrm{b}}$ model. However, for small and intermediate noise magnitudes the prediction of spiral wave tips still works in most cases (e.g. $F_{\mathrm{score}}$ for $\sigma =0.3$: $0.87 \pm 0.021$ ($\mathrm{MM}_{\mathrm{Porc}}$), $0.95 \pm 0.034$ ($\mathrm{BOCF}_{\mathrm{a}}$), $0.979 \pm 0.007$ ($\mathrm{BOCF}_{\mathrm{b}}$), and $0.94 \pm 0.040$ (LR-I), respectively).

### Predicting spiral wave tips of an unknown cell model

**Figure 5:**
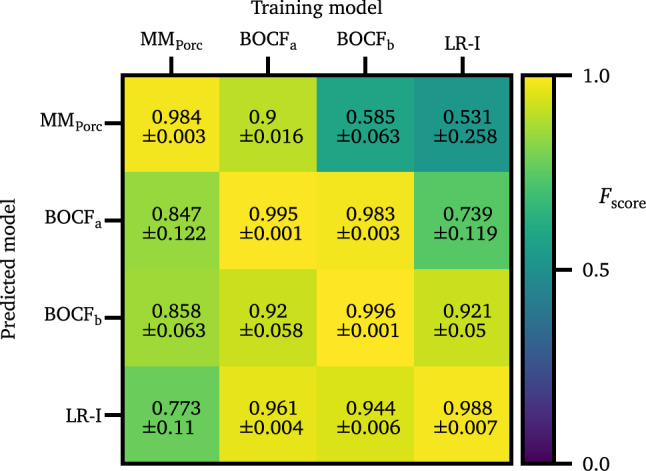
The $F_{\mathrm{score}}$ is shown for all combinations of training models, and prediction models.

How general is the knowledge of the UNet when it is trained with a specific cell model and can it be used to predict the locations of spiral wave tips also for another model? Here, we investigate the objective whether the UNet generalizes to “arbitrary” spiral wave dynamics. For this purpose, we used the UNets which were trained with data from a specific cell model, and predicted the spiral wave tips of another cell model, whose data the UNet has not seen beforehand during the training process. The resulting $F_{\mathrm{score}}$ are shown in Fig. 5.

Although some combinations of training model and prediction model yield good performances (e.g. training with $\mathrm{BOCF}_{\mathrm{a}}$ and predicting LR-I: $F_{\mathrm{score}}=0.961 \pm 0.004$), in average the $F_{\mathrm{score}}$ varies significantly and depends on the specific models (e.g. training with LR-I and predicting $\mathrm{MM}_{\mathrm{Porc}}$: $F_{\mathrm{score}}=0.531 \pm 0.258$). The varying performance of cross-prediction could have different reasons, e.g. different wave shapes (the upstroke of an action potential, for example), different meandering of spiral wave tips, and/or different creation/annihilation processes during the episodes. However, at first sight the $\mathrm{MM}_{\mathrm{Porc}}$ model is the “simplest” model (three dynamical variables) and the LR-I model the most complex one (eight dynamical variables) which we investigated. For future studies it could be interesting to study, whether these features play a major role in the case of cross-prediction.

In a second step, we used data from not only one cell model, but three models for training (still keeping the number of samples to 4000) and tried then to predict the fourth (unknown) model. In this way, we could achieve an $F_{\mathrm{score}}> 0.9$ for each of the four cell models (Table 2). That means, when using several cell models for training and predicting an unknown model, we can achieve a prediction quality that is comparable to the situation where we would have trained with the unknown model itself (comparing Table 2 with Fig. 5).

**Table 2:** This table lists the $F_{\mathrm{score}}$ values for the case, where the UNet was trained with three cell models, and the prediction was performed on the fourth remaining (unknown) cell model.

Training Models	Predicted Model	$F_{\mathrm{score}}$
$\mathrm{BOCF}_{\mathrm{a}}$, $\mathrm{BOCF}_{\mathrm{b}}$, LR-I	$\mathrm{MM}_{\mathrm{Porc}}$	$0.915 \pm 0.02$
$\mathrm{MM}_{\mathrm{Porc}}$, $\mathrm{BOCF}_{\mathrm{b}}$, LR-I	$\mathrm{BOCF}_{\mathrm{a}}$	$0.959 \pm 0.054$
$\mathrm{MM}_{\mathrm{Porc}}$, $\mathrm{BOCF}_{\mathrm{a}}$, LR-I	$\mathrm{BOCF}_{\mathrm{b}}$	$0.989 \pm 0.011$
$\mathrm{MM}_{\mathrm{Porc}}$, $\mathrm{BOCF}_{\mathrm{a}}$, $\mathrm{BOCF}_{\mathrm{b}}$,	LR-I	0.988 ± 0.008

## Discussion

To refer to the three research questions we posed at the beginning of this study, we demonstrate that in general the task of detecting spiral wave tips for different cardiac cell models can be conducted by using deep neural networks. Also, we showed that the algorithm is robust against intermediate noise levels (referring to the second research question). Our answer to the third question is a little more complex: we investigated whether the UNet which was trained e.g. only with model A generalized in such a way, that it can detect also spiral wave tips in model B. We found that the resulting prediction accuracy (measured in terms of the $F_{\mathrm{score}}$) depends significantly on the respective combinations of model A and model B. However, we showed, that if several models are taken into account for training (e.g. model A, model B, and model C) spiral wave tips can reliably be detected for an unknown cell model (model D). This finding suggests, that if the UNet is trained with a pool of different cardiac cell models, it can indeed learn the general concept of what a spiral wave tip is, and can therefore be used in a broad field of applications. For example, a UNet could be trained with a pool of numerical cell models, before it is applied to experimental data (where the ground truth data of positions of spiral wave tips is difficult to access). Possible experimental scenarios for the application of the UNet are ex-vivo Langendorff perfusion experiments^4^ or optogenetic experiments^35,36^.

When comparing conventional methods of detecting phase singularities (which may be computationally demanding) with the application of the UNet approach, a possible speed up in terms of computation time might be essential if the distribution of spiral wave tips must be computed in real-time in an experimental situation. Although, in the first case the actual computation time depends on the specific conventional method chosen, a significant advantage of the UNet approach is that the considerable training time which is necessary can be performed prior to the particular application. However, since the conventional method is computed on CPU, but the UNet approach (implemented with Tensorflow^37^) runs on GPU, comparing computation speeds cannot be done in a rigorous way. Still, with our approach we measure similar speeds for 10000 samples: for the conventional method: $8.41 \pm 0.005$ s (Intel Xeon W-2104 @ 3.20GHz) and the UNet approach (only prediction): $9.11 \pm 0.27$ s (GeForce GTX 1080 Ti). It is noteworthy, that the amount of input data for both methods is different: For the conventional method, two fields (two dynamical variables) per sample are used, whereas in the case of the UNet five fields are required (five delays per sample). Thus, the amount of data per sample is 2.5 times bigger in the case of the UNet. In future studies, where the number of delay vectors or the number of layers, for instance, could be optimized, the UNet approach may be further improved in terms of computation time.

In principle, the approach can also be extended to the task of detecting scroll wave filaments in three-dimensional domains: the detection of (2D) phase singularities in each spatial direction separately could be replaced also here by the UNet. Also, it may be of high interest, whether spiral wave tips can also be detected via a UNet in other systems (e.g. the Belousov–Zhabotinsky reaction).

In the experimental case another advantage of the UNet approach could play a role, if the electrophysiological properties of the system drift with time (non-stationary system)^38^. In this case, also the spatio-temporal dynamics of spiral wave tips can alter significantly. Whereas the parameters of a conventional method might have to be adapted in this situation in order to maintain the functionality of the algorithm, results regarding the $\mathrm{BOCF}_{\mathrm{a}}$ and the $\mathrm{BOCF}_{\mathrm{b}}$ model indicate that the UNet would not need any further adjustments in order to work properly.

In general, this study indicates that the field of research related to the understanding and control of chaotic spiral wave dynamics underlying cardiac arrhythmias could significantly benefit from the application of deep neural networks in many different ways in the future.

## Methods

### Cardiac cell models

More details about the investigated cardiac cell models are given in this section.

#### Porcine model

The $\mathrm{MM}_{\mathrm{Porc}}\, \mathrm{model}$^27^ comprises three variables $(V_{\mathrm{m}}, v, w)$ and the transmembrane currents are given by the sum of a fast inward current $I_{\mathrm{fi}}$, a slow inward current $I_{\mathrm{si}}$, and a slow outward current $I_{\mathrm{so}}$: $I_{\mathrm{tot}} = I_{\mathrm{fi}}(V_{\mathrm{m}}, v) + I_{\mathrm{so}}(V_{\mathrm{m}}, v) + I_{\mathrm{si}}(V_{\mathrm{m}},w)$.

#### Bueno-Orovio–Cherry–Fenton model

The Bueno-Orovio–Cherry–Fenton model^28^ uses four variables ($V_{\mathrm{m}}$, *v*, *w*, *s*) and three transmembrane currents $I_{\mathrm{tot}} = I_{\mathrm{fi}}(V_{\mathrm{m}}, v) + I_{\mathrm{so}}(V_{\mathrm{m}}, v) + I_{\mathrm{si}}(V_{\mathrm{m}},w)$. In this study, we use two different sets of parameters: the $\mathrm{BOCF}_{\mathrm{a}}$ model uses the epicardial parameter set, defined in Table 1 in the original paper^28^, whereas $\mathrm{BOCF}_{\mathrm{b}}$ uses the PB parameter set.

#### Luo-Rudy-I model

The Luo-Rudy-I model^29^ comprises eight variables ($V_{\mathrm{m}}, m, h, j, d, f, X, Ca$) and the transmembrane currents are given by $I_{\mathrm{tot}}=I_{\mathrm{Na}} + I_{\mathrm{si}} + I_{\mathrm{K}} + I_{\mathrm{K1}} + I_{\mathrm{Kp}} + I_{\mathrm{b}}$.

### Numerical integration

All simulations were performed on two-dimensional rectangular simulation domains ($N_x \times N_y = 384 \times 384$ for the $\mathrm{MM}_{\mathrm{Porc}}$ model and $N_x \times N_y = 576 \times 576$ for the $\mathrm{BOCF}_{\mathrm{a}}$ model, $\mathrm{BOCF}_{\mathrm{b}}$ model, and LR-I model), using no-flux boundary conditions. In Table 3 the time step *dt*, the diffusion constant *D* and the grid constant *dx* are given for each cell model.

**Table 3:** This table lists the time step *dt*, the spatial grid parameter *dx* and the diffusion constant *D* for all investigated cell models.

Model	*dt* [$\hbox {ms}$]	*D* [$\hbox {mm}^{2}/\hbox {ms}$]	*dx* [mm]
$\mathrm{MM}_{\mathrm{Porc}}$	0.1	0.1	0.5
$\mathrm{BOCF}_{\mathrm{a}}$	0.1	0.2	0.8
$\mathrm{BOCF}_{\mathrm{b}}$	0.1	0.2	0.8
LR-I	0.1	0.03	0.2

Different chaotic spiral wave episodes were created by initializing a spiral wave, and subsequently perturb the state on random positions. After the application of the perturbation, at least ${4}\,\hbox {s}$ of the episode were discarded, in order to ensure that different chaotic episodes are independent from each other.

### “Conventional” detection of phase calculation

The second dynamical variable (reference variable) and the corresponding reference values which were used to compute spiral wave tips with the conventional method (Eq. (3)) are shown in Table 4.

**Table 4:** This table lists the reference variables and their values for all investigated cell models, which are used to compute the phase in Eq (3).

Model	$V^0_{\mathrm{m}}$	*X*	$X^0$
$\mathrm{MM}_{\mathrm{Porc}}$	0.5	*v* (second variable)	0.1
$\mathrm{BOCF}_{\mathrm{a}}$	0.4	*v* (second variable)	0.1
$\mathrm{BOCF}_{\mathrm{b}}$	0.1	*v* (second variable)	0.1
LR-I	0.4	*j* (fourth variable)	0.2

### UNet architecture

The detailed structure of the UNet (shown in Fig. 2d) is discussed here. The encoder branch of the UNet acts as a feature extractor, alternatingly applying Conv block and Pooling block operations to the input image. A Conv block consists of two 2D convolutional layers with filters of size $3\times 3$, each followed by a batch normalization operation and a tanh activation function. In order to prevent that border pixel are lost, we apply zero-padding prior to the convolution operations. The number of channels (features) is doubled after each Conv block. Each Pooling block encompasses image size reduction by a factor of 2 via Max-pooling and a dropout layer. As a result, 256 features of size $6\times 6$ pixel are extracted in the deepest layer of the UNet.

Along the decoder branch, the extracted features are transformed back to the spatial dimensions of the input image via the alternating application of Up-Conv blocks, skip connections, and Conv blocks. An Up-Conv block contains an up-sampling layer that expands the size of the features by a factor of two and is followed by a 2D convolutional layer with filters of size $2\times 2$. The number of channels (features) is reduced by a factor of 2 after each Up-Conv block, symmetrically with respect to the encoder branch. Skip connections concatenate the output of Up-Conv blocks with their pendants from the encoder branch to enrich the resolution of the up-sampled features and pass the result through a dropout layer. The final Conv-out block, includes a 2D convolutional layer with a single filter of size $1\times 1$, followed by a sigmoid activation function that generates the output mask of the spatial domain. We used binary cross entropy as a loss function.

## Data Availability

The data and the programming code of the UNet that support the findings of this study are available from the corresponding author upon reasonable request.
